# Septic arthritis of the hip, an unreported complication of perforated appendicitis: A case report

**DOI:** 10.1016/j.ijscr.2020.05.093

**Published:** 2020-06-11

**Authors:** Mahnoor Zia, Nima Maghami

**Affiliations:** a Texas A&M College of Medicine, 3050 Health Professions Education Building, 8447 Riverside Pkwy, Bryan, TX 77807, United States; b Houston Methodist Hospital, TAMHSC Partnership, Houston, TX 77030, United States; c Houston Methodist Hospital, Department of Surgery, Houston, TX 77030, United States

**Keywords:** Appendicitis, Interval appendectomy, Arthropathy, Septic arthritis, Arthrocentesis, Case report

## Abstract

**Introduction:**

Progression of inflammation in acute appendicitis can lead to appendiceal perforation, which is associated with higher morbidity and mortality. Bacteremia due to a perforated appendicitis can lead to distal bacterial seeding, resulting in further complications.

**Presentation of case:**

We present here a case of appendiceal perforation in a 32-year-old previously healthy male. Patient was conservatively managed with intravenous (IV) antibiotics and was discharged on hospitalization day-6. Prior to scheduled interval appendectomy, he developed right sided hip pain with decreased range of motion. Computed tomography (CT) scan with contrast showed right hip abscess and enlarged pelvic lymph nodes. Arthrocentesis yielded purulent fluid with leukocytosis (84.8 k/CMM) and neutrophilia (89%). Patient underwent emergent incision and drainage of the hip abscess followed with 42-days of IV antibiotics for septic arthritis.

**Conclusion:**

This case presents a unique occurrence of a complication from appendiceal perforation in patients who are initially managed non-operatively. Clinicians should be mindful of rare complications associated with non-operative management of appendicitis.

## Highlights

• Septic arthritis can result from recent bacteremia.
• Septic arthritis of hip joint secondary to perforated appendicitis has not been previously reported.
• Limited literature exists on complications in patients who are non-surgically managed for perforated appendicitis.

## 1 Introduction

Complicated appendicitis (defined as perforation of the appendix, empyema or abscess formation, and finally fecal peritonitis) is a feared complication of acute appendicitis, associated with higher morbidity and mortality. Progression of inflammation can lead to appendiceal perforations and gangrenous appendicitis which may subsequently progress to peri-appendiceal abscess [1]. Average rate of perforations at presentation are between 16%–30% [2]. A 9% increased risk of appendiceal perforation is reported for each day delay in management [3]. Other risk factors include extremes of age, male sex, febrile on admission, presence of fecalith, immunosuppression, comorbid medical conditions and previous abdominal surgery [3,4]. Postoperative morbidity, reported as high as 20%, is associated with development of abdominal abscess, prolonged ileus, pneumonia, and congestive heart failure [3,5,6].

Timely management of acute perforated appendicitis is necessary, otherwise bacterial seeding can occur. Previously reported rare complications from perforated appendicitis include mesenteric vein thrombosis and necrotizing fasciitis of the thigh [7,8]. We present a unique case of septic arthritis of the hip as a complication of perforated appendicitis. This case illustrates additional complications that can occur due to perforated appendicitis which may need prompt surgical management.

This case report has been reported in accordance with the SCARE Criteria [9].

## 2 Presentation of case

**Fig. 1:**
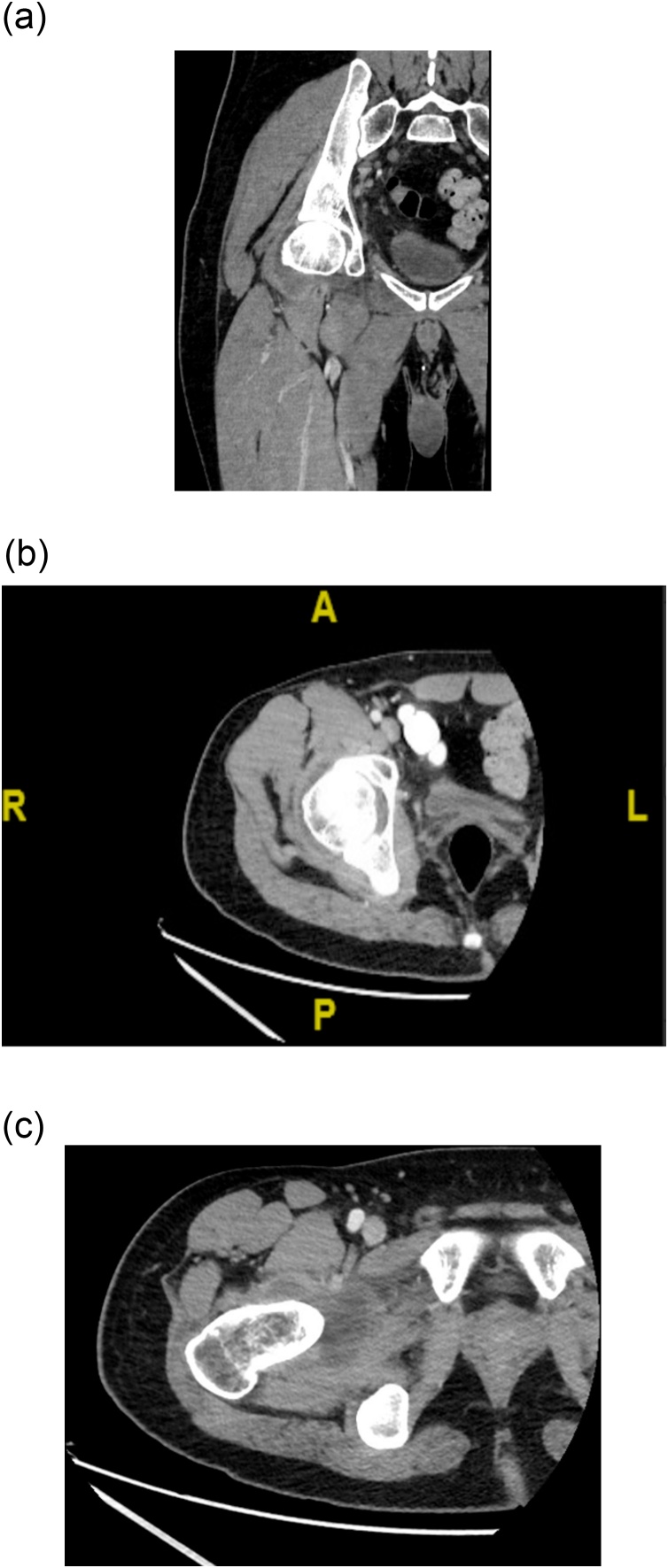
Representative axial and coronal computed tomography (CT) images of the right lower extremity with contrast depicting moderate right hip joint effusion.

**Fig. 2:**
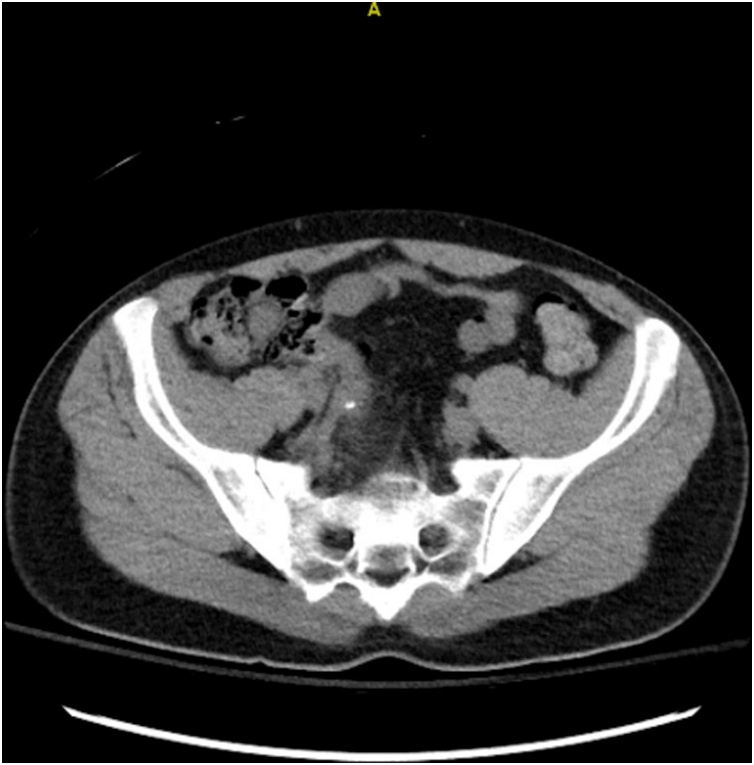
Computed tomography (CT) of abdomen and pelvis showing interval improvement in peri-appendiceal inflammation. Appendicolith is visible at tip of appendix in the right lower quadrant.

A 32-year-old male with a medical history of perforated appendicitis 18-days prior to presentation, was admitted to the hospital with a 4-day history of worsening right hip pain. Physical examination was remarkable for moderate tenderness to palpation on the right lateral hip and restricted range of motion. Laboratory evaluation was notable for elevated inflammatory markers including sedimentation rate (35 mm/hr) and C-reactive protein (7.52 mg/L). Patient was afebrile (98.3 °F) without leukocytosis (7.92 k/μL). Monoarticular joint pain was concerning for septic arthritis or crystal arthropathy. Patient was started on IV vancomycin and cefepime. Per Infectious Diseases recommendations, cefepime was later changed to ertapenem for broader gram negative and anaerobic coverage. Computed tomography (CT) of lower extremities with contrast was remarkable for right hip joint effusion with multiple enlarged right pelvic wall lymph nodes (Fig. 1). CT of abdomen and pelvis showed appendix and previous periappendiceal inflammatory changes had improved to near complete resolution (Fig. 2). Arthrocentesis of the right hip was performed by interventional radiology (IR) and yielded cloudy serous fluid. Synovial fluid analysis revealed leukocytosis (84.8 k/CMM) with neutrophilia (89%) without any evidence of crystals. Bacterial and fungal cultures were negative.

**Fig. 3:**
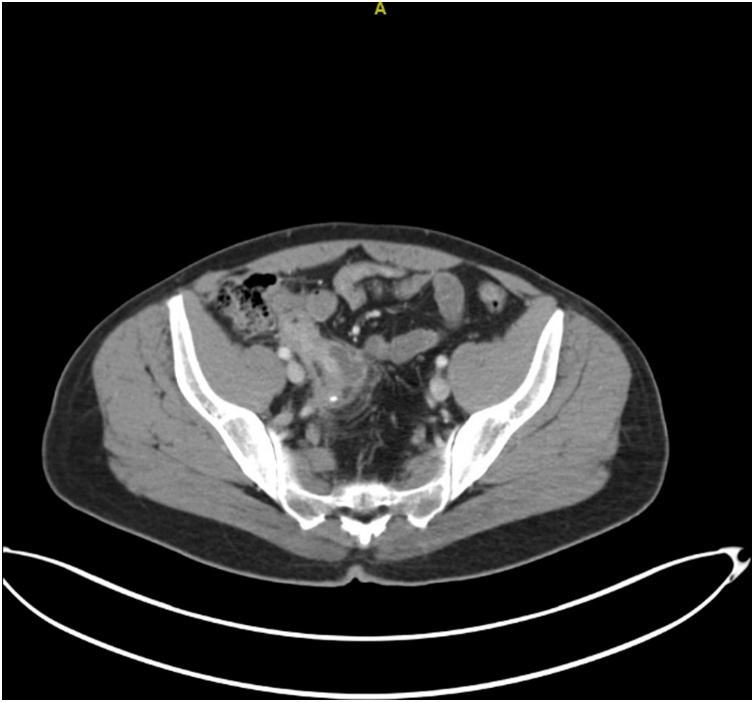
Perforated appendicitis with associated 4.5 cm abscess and surrounding fat stranding and inflammation.

**Fig. 4:**
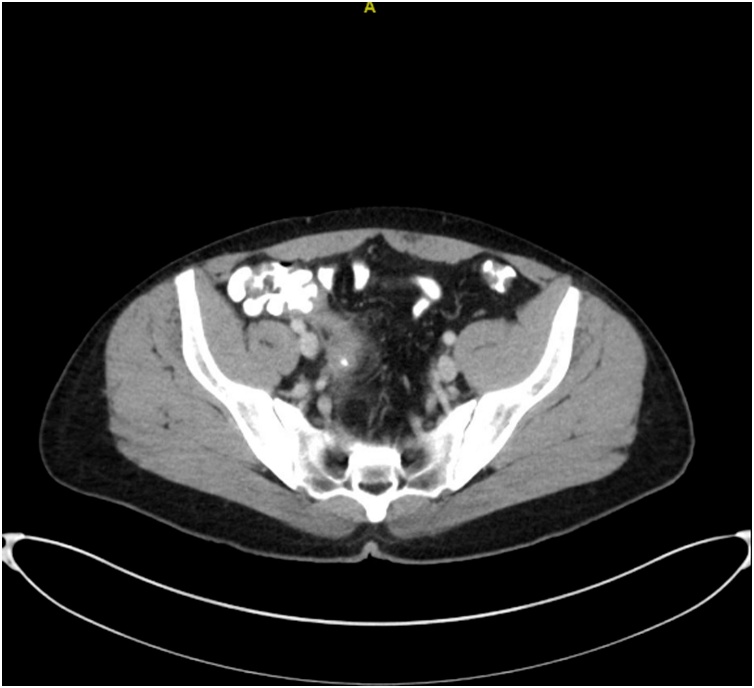
Sequela of perforated appendicitis with interval decrease in size of the previously identified periappendiceal abscess. No drainable fluid collections noted.

On review of past medical history, patient was admitted to our facility 2-weeks earlier with acute onset abdominal pain localized to right lower quadrant, associated with anorexia and a fever (102.3 °F). Serum studies were positive for leukocytosis (14.3 k/uL) with neutrophilia (77%). CT abdomen and pelvis was remarkable for fat stranding and a loculated 4.5-cm fluid collection consistent with peri-appendiceal abscess (Fig. 3). The abscess was too small for IR drainage. Patient was medically managed and started on IV Piperacillin/Tazobactam. Patient stabilized clinically; leukocytosis improved on hospitalization day-2 (7.85 k/μL). Repeat CT abdomen and pelvis on hospitalization day-4 displayed diffusely inflamed appendix with prominent appendicolith at the tip, and the peri-appendiceal abscess had decreased in size to 3-cm and no drainable fluid collection was noted (Fig. 4). Patient was discharged on hospitalization day-6 with oral amoxicillin/clavulanic acid for 14-days. An interval appendectomy was scheduled for 4-weeks post-discharge.

Recovery post hospital admission for perforated appendicitis was complicated with inflammatory arthropathy in the right hip. He underwent an open arthrotomy with incision and drainage of the right hip abscess. Intra-operatively, patient was noted to have copious amount of purulence in the right hip joint, in addition to grade 1 cartilage changes in his right hip, and necrotic right hip capsule. Magnetic resonance imaging of hip without contrast on postoperative day-3 noted post-operative changes, resolution of periarticular abscess, and continued enlarged lymph nodes likely to be reactive. He was discharged with home health and a 42-day course of IV ertapenem.

Upon completion of IV antibiotics, patient returned for an interval appendectomy. Laparoscopic appendectomy was performed. Appendix appeared normal with thin adhesions to the pelvis. Pathology was benign with evidence of acute on chronic inflammatory changes. Patient was seen two weeks post-appendectomy, and he was free of pain, tolerating a diet, and having regular bowel movements. He continued to improve with his ambulation and hip range of motion through a structured physical therapy regimen.

## 3 Discussion

Pathogenesis of septic arthritis involves bacterial invasion of the synovial membrane which leads to inflammatory process producing the characteristic purulent synovial fluid observed with arthrocentesis [10]. Septic arthritis most commonly occurs due to hematogenous seeding secondary to a bacteremia, other causes include penetrating trauma and corticosteroids joint injections [11]. The diagnosis of septic arthritis is established with arthrocentesis of the effected joint. Leukocytosis (WBC of >50k/μL) and polymorphonuclear cells of greater than 90% increase the likelihood of septic arthrits. Diagnosis and etiology are confirmed with gram stain and culture of the joint fluid [11]. Risk factors for septic arthritis includes age older than 60 years, recent bacteremia, degenerative arthritis, rheumatoid arthritis, metabolic syndrome, immune compromised state, joint prosthetics, skin infection and history of sexually transmitted diseases [10,11].

Our patient had presented with monoarticular arthritis, synovial fluid was inflammatory, on open arthrotomy of right hip, extensive purulence was noted. This increased likelihood for septic arthritis. The patient had received IV antibiotics prior to arthrocentesis which can lead to negative bacterial cultures. The only identifiable risk factor for developing septic arthritis in our patient was history of perforated appendicitis. Previously published literature has reported severe complications from complicated appendicitis. For example, hematogenous spread of gut bacteria secondary to appendiceal perforation can lead to mesenteric vein thrombosis and pyogenic liver abscesses [7]. Furthermore, direct propagation of infection through the Petit and Grynfeltt-Lesshaft (inferior and superior lumbar) triangles have led to necrotizing fasciitis of lower limbs [8]. Therefore, several different mechanisms can be responsible for the complication observed in our patient.

## 4 Conclusion

There is a trend to conservatively manage acute appendicitis, this case report highlights a potential complication associated with conservative management of acute appendicitis that clinicians should be vigilant about. The literature is sparse regarding discussion of complications prior to interval appendectomy in patients who undergo conservative management. Septic arthritis due to perforated appendicitis in an immune-competent patient has not been previously reported. This case presentation serves as a unique example of a severe life-threatening complication from a perforated appendicitis, necessitating close follow up with patients who initially present with complicated appendicitis.

## Declaration of Competing Interest

No conflicts of interest regarding this publication.

## Funding

No funds were received for any part of this case report.

## Ethical approval

This case report is exempt from ethical approval at our institution as this is not a research study.

## Consent

Written informed consent was obtained from the patient for publication of this case report and accompanying images. A copy of the written consent is available for review by the Editor-in-Chief of this journal on request.

## Author contribution

Mahnoor Zia: Preparation, creation and presentation of the submission, writing-original draft.

Nima Maghami, MD, FACS: Patient management. Writing, reviewing and editing this submission.

## Registration of research studies

1. Name of the registry: Not required.
2. Unique identifying number or registration ID: not required.
3. Hyperlink to your specific registration (must be publicly accessible and will be checked):

## Guarantor

Nima Maghami.

## Provenance and peer review

Not commissioned, externally peer-reviewed.
